# Notes and News

**Published:** 1904-03-26

**Authors:** 


### NOTES AND NEWS.

The annual festival dinner in aid of the London Orphan Asylum, Watford, will be held at the Hôtel Métropole on Thursday, April 14, when J. C. Parkinson, Esq., J.P., will preside.

Sir Donald Currie has given £100,000 to University College Hospital. The larger portion of this sum is to be devoted to the purpose of enabling the College to amalgamate with the London University.

In the Charter recently granted to the Manchester University it is provided that Associates of Owens College at the date of the Charter shall be offered Bachelors' degrees at the University. At the recent conferment of degrees at the University about 100 Associates presented themselves for graduation.

The Royal Orthopædic Hospital vacates its present premises in Oxford Street and Hanover Square on Lady-day. The Royal Orthopædic and the National Orthopædic Hospitals are to amalgamate, and a new Hospital will be built on the site of the National Orthopædic Hospital in Great Portland Street, with accommodation for 200 patients. A branch hospital for 100 patients will be established in North London.

The hospitals attached to the University of Tomsk, have offered to place twelve beds at the disposal of the Government during the summer for the use of the wounded in the Far East, the patients to be attended by University professors. The military authorities will also have the use of the two buildings in which the students reside, of the first accommodating 70 persons, until the beginning of the next scholastic year, and of the second, arranged for 150 men, for the duration of the war.

It is stated that several cases of smallpox have occurred amongst the in-patients of the London Hospital.

The Hon. W. J. Pirrie has intimated his intention to free the Belfast Royal Victoria Hospital from the debt upon the building. He has already given large sums to the Institution.

A medical man at Wandsworth has been summoned on an accusation of obtaining money from the Wandsworth and Clapham Guardians by means of false representations as to vaccinations performed by him.

Lord Lytton and a Committee are endeavouring to raise funds to establish a Home of Recovery in the country, where surgical patients requiring more medical care and nursing than is provided in convalescent homes, can be received from the hospitals. The locality chosen is Surrey, and the sum asked for is £35,000 to secure 21 beds.

The University of Aberdeen has conferred the honorary degree of Doctor of Laws upon Dr. Cullingworth, Obstetric Physician to St. Thomas's Hospital. The University of Glasgow has conferred the same degree upon Dr. William Stirling, Professor of Physiology, Manchester, and Sir William Taylor, K.C.B., the Director-General of the Army Medical Service.

Mr. Brudenell Carter delivered an interesting and important lecture last week on Physiology and Education, at Owens College, Manchester. In considering the question of the effect of school-work upon under-fed children, he remarked that the question which had not been answered was whether an under-fed child could be taught without sustaining injury in the process? If it could, the State might properly insist upon the teaching. If it could not, the State had no right to inflict the injury.

An outbreak of Plague has occurred at Johannesburg, eight deaths having been reported amongst the coolie population, and the patients number upwards of 40. Drastic measures are being taken to prevent the spread of the disease, and it is held that the climatic conditions are very unfavourable to any serious outbreak. Dr. Marais, who attended upon the first cases, and his wife have died of the disease, and his three children are now dangerously ill. A case of Plague has occurred at Port Said.

The British Medical Association holds its seventy-second annual at Oxford this year in July. The exhibition is to be held in the Examination Schools in the High Street, which are splendid quarters for the purpose. Oxford is a very interesting location for the meeting from the visitors' point of view, but there are drawbacks. The term being over, the members of the University will mostly be away, and to those of the medical profession who have received their education there, Oxford in vacation will not appeal.

